# Dumbbell-shaped thrombectomy device for cerebral venous sinus thrombus removal with controllable axial and longitudinal maneuverability

**DOI:** 10.1093/nsr/nwaf015

**Published:** 2025-01-14

**Authors:** Ming Li, Baoying Song, Yan Wu, Yang Zhang, Xiaofeng Cao, Hongkang Zhang, Yi Xu, Chuanjie Wu, Chuanhui Li, Chen Zhou, Lu Liu, Feng Yan, Sijie Li, Jian Chen, Ran Meng, Jiangang Duan, Di Wu, Lin Zuo, Zikai Xu, Zhou Li, Yufeng Zheng, Miaowen Jiang, Xunming Ji

**Affiliations:** China-America Institute of Neuroscience and Beijing Institute of Geriatrics, Xuanwu Hospital, Capital Medical University, Beijing 100053, China; Department of Neurosurgery and Neuroscience, Xuanwu Hospital, Capital Medical University, Beijing 100053, China; China-America Institute of Neuroscience and Beijing Institute of Geriatrics, Xuanwu Hospital, Capital Medical University, Beijing 100053, China; Department of Neurosurgery and Neuroscience, Xuanwu Hospital, Capital Medical University, Beijing 100053, China; China-America Institute of Neuroscience and Beijing Institute of Geriatrics, Xuanwu Hospital, Capital Medical University, Beijing 100053, China; Department of Neurosurgery and Neuroscience, Xuanwu Hospital, Capital Medical University, Beijing 100053, China; Beijing Institute of Brain Disorders, Capital Medical University, Beijing 100069, China; School of Materials Science and Engineering, Peking University, Beijing 100871, China; School of Materials Science and Engineering, Peking University, Beijing 100871, China; China-America Institute of Neuroscience and Beijing Institute of Geriatrics, Xuanwu Hospital, Capital Medical University, Beijing 100053, China; Department of Neurosurgery and Neuroscience, Xuanwu Hospital, Capital Medical University, Beijing 100053, China; China-America Institute of Neuroscience and Beijing Institute of Geriatrics, Xuanwu Hospital, Capital Medical University, Beijing 100053, China; Department of Neurosurgery and Neuroscience, Xuanwu Hospital, Capital Medical University, Beijing 100053, China; Department of Neurosurgery and Neuroscience, Xuanwu Hospital, Capital Medical University, Beijing 100053, China; Beijing Institute of Brain Disorders, Capital Medical University, Beijing 100069, China; Department of Neurosurgery and Neuroscience, Xuanwu Hospital, Capital Medical University, Beijing 100053, China; Department of Neurosurgery and Neuroscience, Xuanwu Hospital, Capital Medical University, Beijing 100053, China; China-America Institute of Neuroscience and Beijing Institute of Geriatrics, Xuanwu Hospital, Capital Medical University, Beijing 100053, China; Department of Neurosurgery and Neuroscience, Xuanwu Hospital, Capital Medical University, Beijing 100053, China; Department of Neurosurgery and Neuroscience, Xuanwu Hospital, Capital Medical University, Beijing 100053, China; Department of Neurosurgery and Neuroscience, Xuanwu Hospital, Capital Medical University, Beijing 100053, China; China-America Institute of Neuroscience and Beijing Institute of Geriatrics, Xuanwu Hospital, Capital Medical University, Beijing 100053, China; School of Bioengineering, Beihang University, Beijing 100191, China; School of Life Science, University of Glasgow, Glasgow G12 8QQ, Scotland; CAS Center for Excellence in Nanoscience, Beijing Key Laboratory of Micro-Nano Energy and Sensor, Beijing Institute of Nanoenergy and Nanosystems, Chinese Academy of Sciences, Beijing 100083, China; Tsinghua Changgung Hospital, School of Clinical Medicine, School of Biomedical Engineering, Tsinghua Medicine, Tsinghua University, Beijing 100084, China; School of Materials Science and Engineering, Peking University, Beijing 100871, China; Beijing Institute of Brain Disorders, Capital Medical University, Beijing 100069, China; China-America Institute of Neuroscience and Beijing Institute of Geriatrics, Xuanwu Hospital, Capital Medical University, Beijing 100053, China; Department of Neurosurgery and Neuroscience, Xuanwu Hospital, Capital Medical University, Beijing 100053, China; Beijing Institute of Brain Disorders, Capital Medical University, Beijing 100069, China

**Keywords:** NiTi stent retriever, biomechanical compatibility, cerebral venous sinus thrombosis, thrombus removal

## Abstract

Cerebral venous sinus thrombosis (CVST) is frequently observed in younger adults and features in large thrombus volume. Due to the triangular-like cross-sectional shape and large diameter of the superior sagittal sinus, all the commercially available artery stent retrievers are not suitable for venous vessels. In this study, a dumbbell-like stent was designed and fabricated by 3D braided technology using NiTi wires; it was manually rotatable and stretchable with controlled length/diameter ratios (2.6–14.0) and reciprocating maneuverability. Computational modeling and an *in vitro* study were conducted to evaluate the mechanical properties of this device and its ability to trap and remove thrombi from occluded venous vessels was verified by using a swine model. A single-center retrospective clinical study of 10 patients using the Venus-TD to treat patients with CVST was also conducted. Pre/postoperative thrombus volume in 10 patients was quantitatively analysed (12 855.3 ± 6417.1 vs. 2373.1 ± 2759.0 mm³, *P* < 0.001) with a high recanalization rate, yielding favorable clinical outcomes. This study offers a novel treatment option for patients with extensive CVST.

## Teaser

This work presents a novel dumbbell-shaped stent designed for treating extensive cerebral venous sinus thrombosis with good biomechanical compatibility, offering improved thrombus removal and promising clinical outcomes.

## INTRODUCTION

Cerebral venous sinus thrombosis (CVST) is associated with a mortality rate of 5%–10% and mostly affects young people [1]. According to a large prospective cohort study, the mean age at diagnosis was 39 years and three-quarters of the patients were female [2]; 5%–20% of all CVST cases were associated with pregnancy and puerperium [3]. For patients with severe and anticoagulant-refractory CVST, endovascular treatment (EVT) can be beneficial for clot dislodgement and removal [4]. However, a recent randomized clinical trial (TO-ACT) evaluating EVT in patients with severe cerebral venous stroke was prematurely terminated because of futility. One explanation was the significant difference between EVT in the venous and arterial systems. During enrollment, existing technologies and devices for optimal recanalization in CVST patients are inadequate and novel devices that are capable of faster and more effective thrombus removal from the cerebral venous system are lacking [5]. Therefore, it is of significant importance to make dedicated stent retrievers for safe and effective thrombectomy in CVST [4,6].

The application of EVT in cases of CVST differs significantly from that of arterial ischemic stroke in terms of anatomical structure and thrombus pathology (Table S1). First, cerebral venous sinuses can be easily damaged during mechanical thrombectomy due to the presence of arachnoid granules and fibrous cords (that exist in the form of lamellar, trabecular and valve-like shapes) [7,8] and this complication can lead to new thrombosis in the sinuses [9]. Second, the shape of the intracranial venous vessel lumen is irregular (e.g. triangular) and the luminal diameter varies greatly, ranging from 6–13 to 15 mm. However, the diameters of the available aspiration catheters and stent retrievers are <6 mm and, thus, they cannot be utilized for complete thrombus removal [4]. Third, the sinus wall lacks the smooth muscle that is found in arteries and has inelastic arachnoid granulations, posing a significant risk of iatrogenic hemorrhage during the intervention and the initiation of new thrombosis. Fourth, the intracranial sinus system is more tortuous, especially for junction segments such as that between the sigmoid sinus and the jugular vein. Off-label use of arterial stent retrievers poses a high risk of buckling within these segments with acute anatomic curvature, perforation of venous sinuses and vessel dissection [10]. Fifth, the venous clot burden is usually high, as the sinus caliber is much larger than those of arteries. According to Machi *et al.*, regular stent retrievers are ineffective for white thrombi with large diameters of >6 mm [11]. Therefore, a dedicated stent retriever for cerebral venous sinus thrombosis is necessary for rapid and efficient recanalization.

Currently available intracranial retrievable stents for acute ischemic stroke (AIS) treatment are made of NiTi alloys by using laser-cutting technology, including Solitaire (Medtronic) and Trevo (Concentric Medical) stents [12]. In addition to the caliber discrepancy between the devices and venous vasculature, another major concern is the risk of wall injury induced by laser-cut stent expansion and clot retrieval [13]. As an alternative, braided stents may provide lower radial force and can potentially minimize the associated vessel damage. Recently, a novel manually expandable stent retriever (Tigertriever) for AIS was fabricated with a braided mesh consisting of NiTi wires [14], which provides a customized adjustment of radial force for various thrombus and vessel conditions [15]. A prospective multicenter study using Tigertriever for large vessel occlusion AIS indicated a good final reperfusion rate (94%) that was higher than those of other common thrombectomy devices [15].

In view of the unique physiological structure of cerebral veins, the present study developed a dedicated venous sinus thrombectomy device (Venus-TD) by 3D braided technology using NiTi wires, which is manually rotatable and stretchable with controlled length/diameter and reciprocating maneuverability. Computational modeling and an *in vitro* study were conducted to evaluate the mechanical properties of this device and the ability to trap and remove thrombi from occluded venous vessels was verified by using a swine model. Finally, a single-center retrospective clinical study using the Venus-TD to treat patients with CVST was conducted.

## RESULTS

### Stent design and demonstration

**Figure 1.**
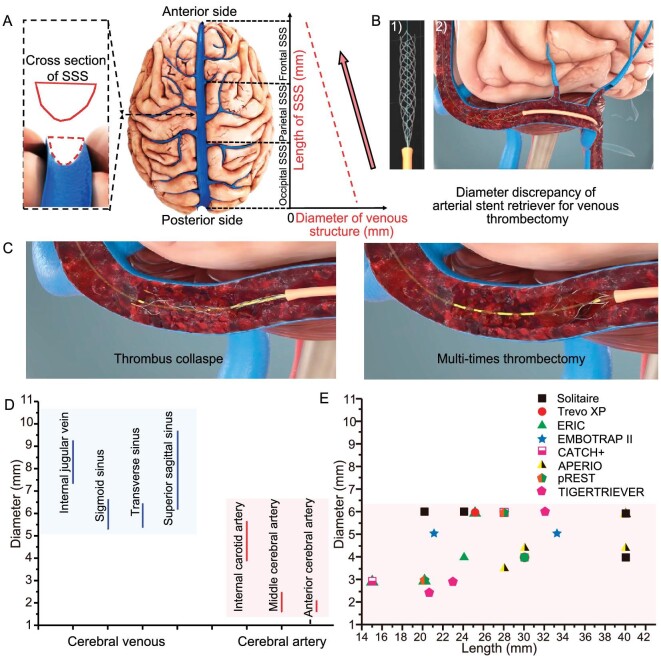
Illustration of the physiological structure of cerebral veins. (A) Cross-sectional view of the superior sagittal sinus (SSS) and its diameter changes. Arachnoid granulations and chordae (including lamellar, trabecular and valve-like types) within the SSS are not depicted in Fig. 1 and typical photos of these interior structures can be found in [7,8]. (B) Demonstration of thrombus removal by using a typical arterial stent retriever and its disadvantages, such as (C) thrombus collapse and the need for multi-time thrombectomy. (D) Diameter ranges of typical cerebral venous and artery vessels (Table S2) [37,44–46]. (E) Comparison of diameter/length values of current commercially available stent retrievers for cerebral artery occlusions (Table S3) [47].

The anatomical structure of the cerebral venous system varies greatly in comparison with the arterial system. The typical vertex view of the superior sagittal sinus (SSS) is shown in Fig. 1A. The average width of the SSS in the mid-anterior frontal region was 4.3 mm and there is, on average, a mean width of 9.9 mm in the mid-occipital region [16]. Hence, the SSS is narrow anteriorly and wide posteriorly. In addition, the cross-sectional shape of the SSS lumen was triangular with its apex pointing inferiorly and, compared with the internal carotid artery (ICA), the internal jugular vein (IJV) displayed an elliptical-like cross section. The SSS is the most common location of CVST, which is difficult to access with the currently available endovascular tools. The utilization of an arterial stent retriever (e.g. a Solitaire stent) may lead to thrombus collapse and multi-times thrombectomy (Fig. 1B and C). Therefore, better conformability and flexibility of the stent are required to enable the safe and efficient removal of thrombi. As shown in Fig. 1D and E, as most of the current devices are developed for intracranial artery vessels and are not consistent with the cerebral venous sinus (CVS) caliber, it may not achieve good thrombus removal and vascular recanalization effects.

**Figure 2.**
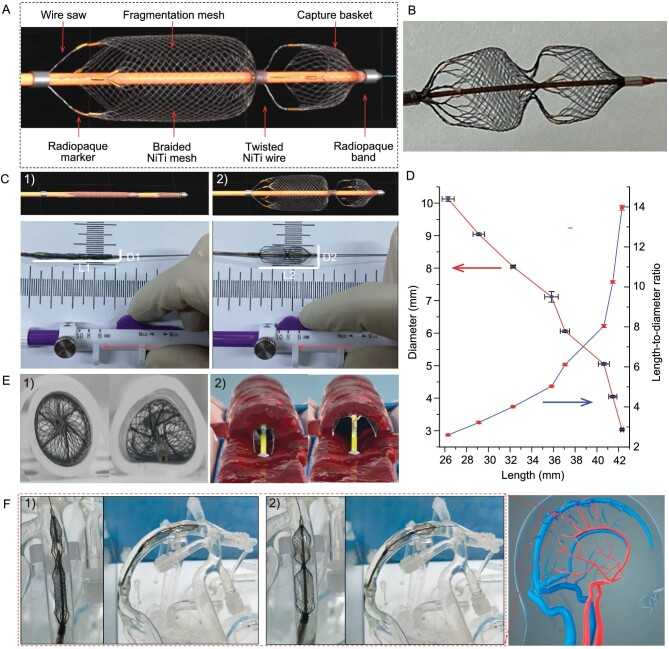
Demonstration of the Venus-TD. (A) Design and structure of the device. (B) Photo of the device. (C) Photos of the geometrical changes of the device with a controllable length/diameter ratio. (D) Changes of the stent diameters in different lengths, as well as the related length-to-diameter ratios. (E) Photos of stent apposition in silicone tubes with (1) typical geometric cross sections and (2) illustration of transverse fragmentation of the thrombus. (F) Simulated delivery of the device into the SSS within the cerebral venous models under (1) elongated and (2) expanded states.

The proposed Venus-TD is a novel NiTi wire-braided and manually adjustable stent for fragmenting and removing clots, and consists of a thrombus capture basket, fragmentation mesh and wire saw. The stent was designed with two stainless steel radiopaque bands on both sides and eight gilded tungsten wires as radiopaque markers, forming a dumbbell-like shape (Fig. 2A and B). The technical concept of a controllable length/diameter ratio (*L*_1_/*D*_1_ > *L*_2_/*D*_2_) is realized by connecting the NiTi stent to coaxially inserted microwires that are fixed to the proximal control handle (Fig. 2C). By pulling or pushing the handle, the stent length can range from ∼42 to 26 mm and the diameter from 3 to 10 mm (Fig. 2D). These modifications can be set continuously by the operator, which could potentially enable the stent to have versatile flexibility to better accommodate the cerebral venous vascular calibers. The device also displayed good conformability and close apposition to the deformed silicone tubes, which represented mock vascular vessels (Fig. 2E(1)). As illustrated in Fig. 2E(2), by pulling or pushing the handle, the stent can be manipulated in different configurations to facilitate the axial penetration and fragmentation of the clot. The feasibility to deliver the Venus-TD was determined by using a mock venous system (Fig. S1). This thrombectomy device was easily navigated into the SSS in the phantom study and manipulated into an elongated (*L*_1_/*D*_1_) and expanded (*L*_2_/*D*_2_) state (Fig. 2F and Fig. S2). An animation video that demonstrates the thrombectomy process is provided as Video S1.

### Computational modeling of stent and its interaction with venous walls

**Figure 3.**
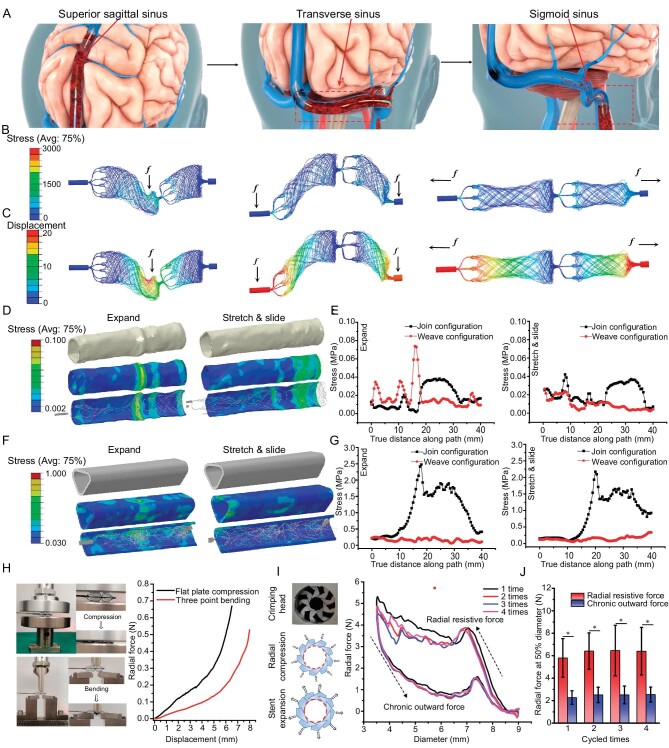
Mechanical evaluation of the Venus-TD. (A) Schematic illustration of thrombus removal by using the Venus-TD from the superior sagittal sinus (SSS), transverse sinus (TS) and sigmoid sinus (SS). (B) The von Mises stress and (C) the displacement contour plots from computational modeling of the devices with weave configurations under different mechanical scenarios. The von Mises stress distribution on the internal jugular vein (IJV) (D and E) and SSS (F and G) vessels walls induced by the expanding and stretching/sliding of the device, as well as the related values along the vessels compared with join configuration. (H) Photos and force-displacement curves of the stent under flat plate compression and three-point bending. (I) Photos of the crimping head and schematics of the radial compression and stent expansion. The circumferential radial force-stent diameter curves of the stent after four load–unloading cycles. (J) The radial force of the stent that had been compressed to 50% of its labeled diameter was recorded at different cycles. The related point position is depicted in Fig. S9. *P < 0.05.

Biomechanical compatibility between the stent and the venous wall is of significant importance during the retrieval process. The stent should be flexible to allow safe navigation through the acute angulation, such as the junction between the SSS and the transverse sinus (TS) and sigmoid sinus (SS) segment (Fig. 3A). The whole process was initiated from the distal to the proximal end of the sinus in a step-wise approach until the sinus that was affected by thrombus was recanalized.

Laser cutting is a commonly used approach to make stents or stent retrievers [17]. Compared with laser-cut stents, the wire-braided counterparts exhibited higher flexibility due to the capacity of individual wires to slide and rotate around each other. To further illustrate the importance of this feature, a join-configurational structure was used as a control group and their mechanical properties were compared by using a computational modeling method (Figs S3 and S4).

The von Mises stress and displacement contour plots of the weave (Fig. 3B and C) and join (Fig. S5) configuration devices under bending and stretching are modeled. No obvious high-stress zones were produced and the quantitative results indicated comparable mechanical properties of both thrombectomy devices (Fig. S6). The join-configurational braided stent displayed higher displacement values than the weave-configurational braided stent and this feature suggested that the braided Venus-TD stent was more flexible and had higher biomechanical compatibility with vessel walls.

The Venus-TD was deployed by using a transjugular approach with the guiding wire and catheter, and was advanced to the distal side of the target thrombus; meanwhile, aspiration was performed through the coaxially inserted catheter by using an autotransfusion system (Fig. S7). The thrombectomy process mainly consisted of stent compression, release (expand) and retrieval (stretch and slide) (Videos S2 and S3).

The utilization of a flexible stent can reduce the risk of perforation (cerebral hemorrhage) and mechanical injury (intimal injury that causes vascular restenosis). Therefore, given the unique structure of cerebral venous vessels, the IJV and SSS were selected as target vessels. The stress distribution that was induced by the expanding and sliding of the stent on the inner IJV and SSS walls was also simulated and analysed by using computational modeling. The deformed vessel geometry that was induced by the expanding and stretching/sliding of the weave-configurational braided Venus-TD is shown in Fig. 3D and F, as well as that of the control group (Fig. S8). Visualization of the numerical results clearly showed that, compared with the join structure, no high-stress concentration zones were observed on both vessel walls that were induced by the flexible weave structures. The join-configurational braided stent displayed higher displacement values than the weave-configurational braided stent (Fig. 3E and G, and Fig. S9) and this feature suggested that the braided Venus-TD stent was more flexible and had higher biomechanical compatibility with vessel walls. This discrepancy was also much larger in SSS.

### *In vitro* mechanical evaluation

Thrombus retrieval efficiency is influenced by multiple factors and stent radial force and flexibility are of great significance. The force obtained at the 4.5-mm displacement of the loading pin in the flat plate compression test was 0.2 N and increased to 0.65 N at a 6.5-mm loading length (Fig. 3H). The force displacement of the three-point bending test displayed a similar trend. To further obtain the mechanical responses of the Venus-TD under different configurations, circumferential radial forces of the stent were measured by using a nine-plate crimping head, as illustrated in Fig. 3I. Both the radial resistive force (the compressing force used to crimp the stent, *F*_rr_) and the chronic outward force (the restoring force used on the stent to expand, *F*_co_) were recorded. The radial resistive force refers to the force that is required to compress the stent radially, while the chronic outward force is the force that the stent exerts on the vessel during expansion. The stent is crimped up to a nominal outer diameter of 2.0 mm. During repeated measurements, the maximum radial resistive force was obtained and the chronic outward force increased with decreasing stent diameters. The radial forces were measured when the device was compressed to 50% of its labeled diameter at each cycle (Fig. 3J). The radial forces of the stent in the resistive state and outward expansion state were ∼5.83 ± 1.81 and ∼2.34 ± 0.65 N, respectively. These results indicated the flexibility of the designed stent and its biomechanical compatibility with the venous vessel walls.

### Animal study

Biocompatibility of NiTi-based medical devices is a major concern of the scientific community. Prior to conducting an animal study, the *in vitro* cytotoxicity of the device was also evaluated. The inductively coupled plasma test results shown in Fig. S10 demonstrate that no Ni ions were detected in the extract or that the Ni ions were lower than the detection limit. The *in vitro* human umbilical vein endothelial cells experiment, as shown in Fig. S11, suggests that the NiTi wire does not have cytotoxicity caused by short-term dissolution of Ni ions and heat treatment will not affect the biocompatibility of the material.

**Figure 4.**
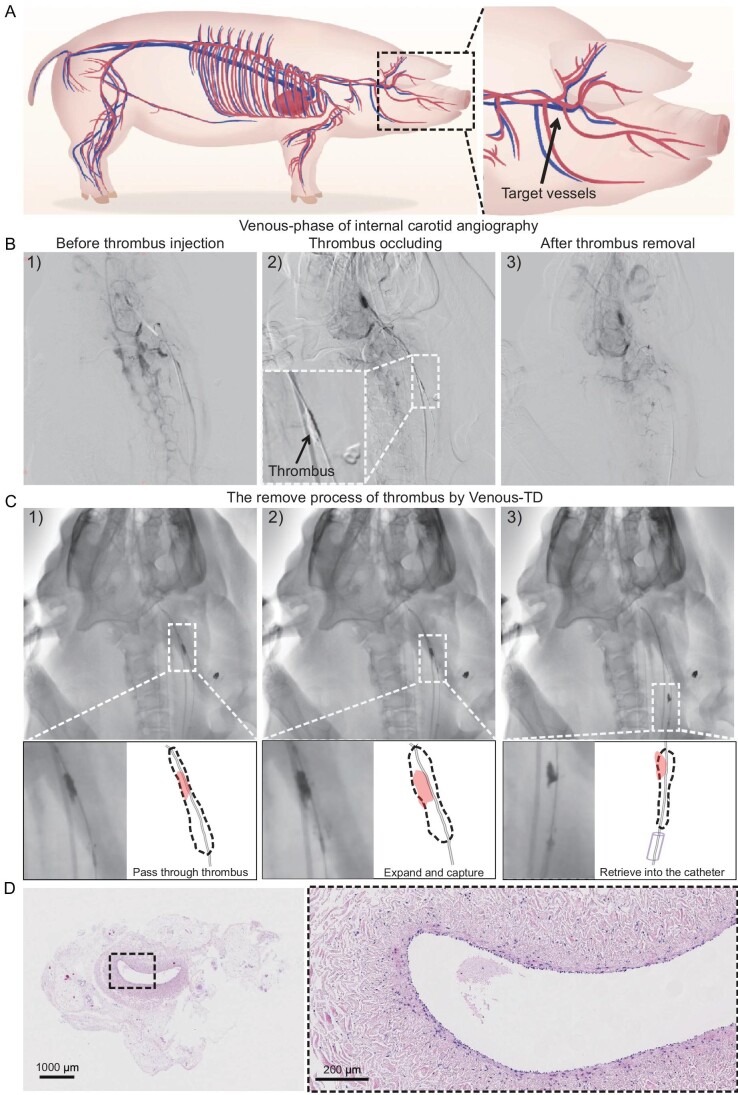
Thrombectomy process in swine models. (A) Illustration of swine cerebral vessels and the targeted jugular veins for thrombus occlusion. (B) The modeling and thrombectomy processes were evaluated by using the venous phase of the digital subtraction angiography (DSA) images of the swine left IJVs (1) before the thrombus injection, (2) after the thrombus occlusion and (3) after thrombus removal. (C) Detailed demonstration of the thrombectomy process by using DSA images: (1) passed through the thrombus, (2) expanded to capture the thrombus and (3) retrieved into the catheter. (D) The hematoxylin–eosin staining images of the IJV.

Swine models were utilized to evaluate the feasibility of the Venus-TD for thrombus removal before testing this device in a clinical setting. The anatomy of the swine cerebral venous system is shown in Fig. 4A. All procedures were performed on animals under general anesthesia with continuous vital-sign monitoring. A balloon catheter (Boston Sterling φ6 mm or Aviator Plus φ6 mm) was navigated into the common jugular vein. The balloon was inflated and contrast injections via the microcatheter confirmed occlusion of the sinus. After confirmation, a volume of 10–12 mL of visualized thrombus was injected through the microcatheter while the balloon remained inflated. After 15 min, the balloon was deflated and evacuated (Fig. 4B).

Endovascular thrombectomy was performed after 1 h of cerebral venous occlusion. An 8F guide catheter (Penumbra Neuron MAX 088) was placed at the proximal end of the thrombus. The Venus-TD passed through the thrombus along the 0.014-long microguide wire and reached the distal end of the thrombus (Fig. 4C(1)). The thrombectomy device was adjusted to the appropriate working diameter to match the diameter of the modeled vessel site and to begin capturing the thrombus (Fig. 4C(2)). At this time, the 8F guiding catheter was connected to the syringe for negative pressure aspiration and the thrombectomy device was gradually and mechanically fragmented and removed from the distal to the proximal end of the thrombus until the thrombus was cleared and the vessel was recanalized. The device with the thrombus was subsequently retrieved into the guide catheter (Fig. 4C(3) and Video S4). Arteriography immediately after thrombectomy revealed that the IJV segment was recanalized, the tortuosity of the intracranial vein was reduced and the retention of the contrast agent was significantly reduced, indicating that the cerebral venous reflux was normal. Endovascular thrombectomy was successfully performed in all five swine and only one thrombectomy operation was performed. All five swine survived with stable vital signs and no angiographic complications, such as vascular perforation, extravasation or dissection, thrombus detachment or distant embolism, were observed during the operation. The swine after the above thrombectomy procedure was euthanized and the IJV on the side of the thrombectomy was sectioned and stained by using hematoxylin–eosin staining. As shown in Fig. 4D, there was no significant disruption of the intima of the vessel after device manipulation and the vessel structure remained relatively intact.

### Clinical case reports

**Figure 5.**
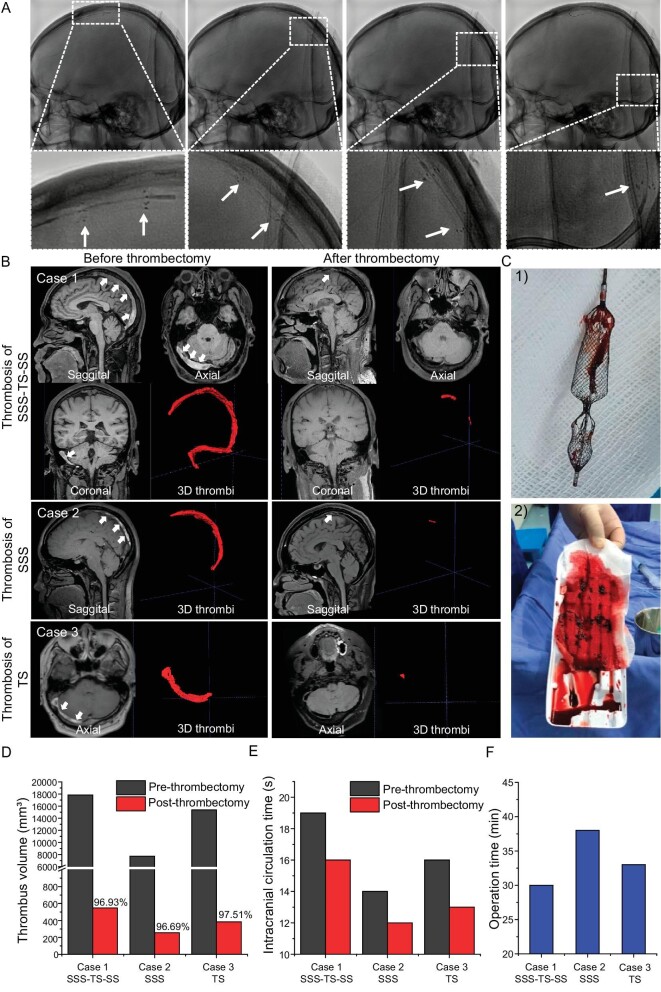
Thrombectomy process in CVST patients. (A) The continuous fragmentation and removal of thrombus along the SSS and torcular herophili are displayed by using DSA and the white arrows indicate the stent markers. (B) For Case 1, the MRBTI demonstrated a isointense and hyperintense mix in the SSS, right TS and SS (white arrowheads) and 3D thrombi were delineated semi-automatically by using software; the followed-up MRBTI after thrombectomy demonstrated complete recanalization of the occluded vessels. Cases 2 and 3 are CVST patients with thrombosis in the SSS and TS, respectively. (C) Optical photos of (1) the retrieved Venus-TD and (2) the removed thrombus from the patient. Their (D) 3D thrombus volume and (E) intracranial circulation time are quantitatively displayed before and after thrombectomy. (F) The operation time of the thrombectomy procedure by using the Venus-TD in these three cases.

For the clinical study, three typical cases of thrombectomy were presented in detail to clarify the thrombectomy process and to perform a quantitative analysis of the thrombus before and after thrombectomy. The feasibility and flexibility of the Venus-TD are demonstrated in Fig. 5A, in which the continuous fragmentation and aspiration of the thrombus along the SSS and torcular herophili were displayed under digital subtraction angiography (DSA) and the white arrows indicate the stent markers. A real-time thrombus retrieval video is provided as Video S7. The thrombus volume was calculated by using magnetic resonance black-blood thrombus imaging (MRBTI) [18].

A 52-year-old male was brought to our hospital emergency service with a history of progressively worsening headache, nausea and vomiting for 2 days. On examination, the Glasgow coma score (GCS) was 15 and the National Institutes of Health Stroke Score (NIHSS) was 0 (Tables S4 and S5). MRBTI showed that the patient had a high-loading thrombus volume (baseline volume of 17 840 mm³) in the superior sagittal, right transverse and sigmoid sinuses (Fig. 5B—Case 1). The venous phase of cerebral angiography showed a filling defect in the thrombosed cerebral vein/sinus, as well as venous congestion with dilated cortical and scalp veins and reversal of venous flow (Fig. S12A and Videos S5 and S6). A mechanical thrombectomy was performed on Day 12 after the symptom onset. A large number of thrombus fragments were retrieved (Fig. 5C). On follow-up angiography, the SSS, right TS and sigmoid sinus were recanalized (Fig. S12B).

The above-mentioned case (Fig. 5B—Case 1) represents a typical and complicated CVST patient with large volumes of thrombus occluded in SSS–TS–SS. The thrombus removal efficiencies were also demonstrated in a CVST patient with thrombosis in SSS (Fig. 5B—Case 2) and TS (Fig. 5B—Case 3). The MRBTI reexamination in all the cases at 1 day after the operation showed complete recanalization of the venous sinus. The quantitative analysis of the residue thrombus for the cases are shown in Fig. 5D and the thrombectomy efficiency was around 96.69%–97.51%. The intracranial circulation time was significantly improved by angiography after thrombectomy (Fig. 5E). The mean thrombectomy procedure time with the Venus-TD device was 33.7 min (Fig. 5F). The patient recovered well and the headache symptoms were significantly improved. The patient was discharged with an mRS score of 1.

### Single-center retrospective study

To further illustrate the efficacy and safety of the Venus-TD, we conducted a retrospective controlled study with a small sample size. A total of 10 patients were included in this study and evaluated by using NIHSS and GCS upon admission; the baseline patient demographics and characteristics are summarized in Table S7. Thrombi were present in a total of 57 segments (10 SSSs, 3 straight sinuses, 7 torcular herophili, 17 TSs, 17 SSs and 3 IJVs).

Semi-automated thrombus volume calculations were performed on all patients before and after the thrombectomy process and the mean procedure time was 33.2 ± 10.6 min. Immediate postoperative angiography revealed that the rate of complete recanalization was 60% (6/10) and the remaining patients (4/10) achieved partial recanalization. There were no significant differences in baseline thrombus volumes between the complete and partial recanalization groups (14 049.0 ± 4391.8 vs. 12 059.5.0 ± 7787.8 mm³, *P* > 0.05). There was a significant difference in the preoperative and postoperative thrombus volumes (12 855.3 ± 6417.1 vs. 2373.1 ± 2759.0 mm³, *P* < 0.001). As shown in Table S8, we quantitatively measured the baseline and residual thrombus volumes of four segments (IJV, SS, TS and SSS). The thrombus clearance rate of the Venus-TD was 77.8% for SSS, 86.4% for TS, 87.3% for SS and 84.2% for IJV. Most residual thrombi were adherent to the walls of the distal SSS and the transverse–sigmoid sinus junction. At the 90-day follow-up, eight patients (80%) recovered without disability with an mRS score of 0–1 and all patients achieved favorable outcomes with an mRS score of 0–2. Major hemorrhagic complications, new symptomatic intracranial hemorrhage (ICH), device-related intraprocedural complications or other serious adverse events were not observed.

## DISCUSSION

In summary, we demonstrated the advantages of the Venus-TD, which is a dumbbell-like 3D braided NiTi stent retriever, as a dedicated venous sinus thrombectomy device for rapid and efficient recanalization, which is manually rotatable and stretchable with controlled length/diameter and reciprocating maneuverability. This device is biomechanically compatible with cerebral venous walls. The efficacy and safety of capturing and removing thrombus from occluded venous sinuses were validated in a swine model. In a cohort of 10 patients, the Venus-TD demonstrated good thrombus clearance capacity and a high recanalization rate via quantitative preoperative/postoperative thrombus volume analysis. Consequently, these patients achieved favorable clinical outcomes.

The EVT of CVST includes balloon catheter thrombectomy and stent thrombectomy. The utilization of balloon catheters may squeeze the thrombus into the adjacent cortical veins to aggravate the disease. The latter one, represented by a Solitaire stent retriever, has been widely used in arterial thrombectomy. The Solitaire stent is a laser-cut stent with a closed-cell design that features enclosed mesh pores and this design provides a robust radial support force but increases the risk of vascular wall injury, particularly in tortuous vessels [13]. Conversely, the Venus-TD is a flexible NiTi wire-braided stent, making it suitable for thrombus removal in tortuous vessels and minimizing associated vascular injuries. As for the thrombectomy procedure, the Solitaire stent allows only one unidirectional thrombectomy. However, as demonstrated in Videos S2 and S3, the Venus-TD features manually controllable length/diameter changes and reciprocating operability, which could be an alternative to the Solitaire stent.

The length and diameter of a stent retriever play a crucial role in determining its effectiveness. Studies have shown that the use of a longer stent retriever with a larger nominal diameter can significantly improve the overall success rates of the procedure [19]. Specifically, stent retrievers with extended lengths provide a greater working capacity, thereby potentially allowing enhanced integration of the device within the clot and even distribution of forces during traction [20]. Stent retrievers with larger diameters also come with higher radial force and better vessel wall apposition [19]. It was reported that the double stent-retriever technique was efficient at enhancing the rates of recanalization on the first pass [21,22]. In our study, the dumbbell-shaped thrombectomy device (Fig. 2A) also may have improved the efficiency of cutting and removing the thrombi. Compared with a stent retriever with the same length and diameter (but without the dumbbell structure, as shown in Fig. S13A), the thrombectomy device with the dumbbell configuration was more mechanically flexible in passing through the vessels with acute anatomic curvature, reducing the mechanical stress on the vessel wall (Fig. S13B).

Pilot clinical studies were conducted in this paper to provide real-world evidence about the clot-removal performance of the novel Venus-TD. The recanalization was achieved in 10 patients and no complications, such as new ICH or embolism, were observed in our study. A high recanalization grade of CVST is independently associated with good neurological outcomes [23]. In 2015, Siddiqui *et al.* conducted a systematic review comprising 185 patients undergoing endovascular thrombectomy for medically refractory CVST; overall, 74% of the patients had near complete recanalization and 10% experienced new or increased ICH [24]. In another systematic review, conducted by Ilyas *et al.* in 2017, that comprised 235 patients, complete radiographic resolution of CVST was achieved in 69% of cases and worsening or new ICH occurred in 8.7% of cases; otherwise, 6.3% of cases had other complications [25]. In 2019, Lewis *et al.* conducted a systematic review and meta-analysis that included 116 patients undergoing intra-arterial/intrasinus chemical thrombolytics combined with mechanical thrombolysis; the complete recanalization rate was 75% and the postprocedural hemorrhagic rate was 17% [26]. As for the operation time, the reported mean procedural time required for endovascular thrombectomy ranged from 89 to 210 min (Table S6 [5,27–34]). Compared with traditional thrombectomy devices (e.g. the Solitaire stent), the Venus-TD had a shorter procedure time (33.7 min).

In our study, we used swine to establish an IJV thrombosis model; the swine anatomy bears a close resemblance to that of humans, making it suitable for studying the pathophysiological mechanism and treatment of CVST. One exception is that the CVS in swine mainly drains into the spinal venous plexus but not the IJV [35]. The incidence of thrombosis varies according to the location of the CVS and the SSS is the most commonly affected sinus (62%) [2]. A study was conducted to evaluate the efficacy and safety of the Trevo XP stent retriever by using a swine model with SSS thrombosis [36]. However, the CVST model in swine is not stable because the vascular structure of the CVS in swine is prone to variation. Moreover, MRV studies of the CVS in swine have revealed that the mean diameter of the SSS is 2.4 ± 0.56 mm; however, the mean diameter of the SSS in humans is 6.2 mm (ranging from 4.1 to 8.3 mm) at bregma [37,38]. It is difficult for the Venus-TD to retrograde into the SSS through the ICA and it is easy to damage the inner vessel walls. At present, the porcine carotid artery is typically used as the target vessel to evaluate the safety and efficiency of the stent, which is important to establish in preclinical research [39–41]. Taking the above factors into consideration, we used the swine jugular veins as the target vessels.

Our study initially confirmed the efficacy and safety of the novel thrombectomy device in patients with CVST. Nevertheless, our clinical study has several limitations. First, as a retrospective study, there was no control group of conventional anticoagulant therapy for CVST patients and only 10 patients were included, which made it difficult to conduct subgroup analysis and statistically compare the efficacy of EVT with novel thrombectomy devices and standard treatment. Another limitation is that the study was a single-center retrospective study, representing a lower level of evidence in the context of evidence-based medicine. To further confirm the effectiveness of EVT with this device, multicenter randomized–controlled trials with larger samples are urgently needed.

## METHODS

### Stent manufacture

NiTi wires (50.7 at.% Ni) with a diameter of 0.06 mm were purchased from Fort Wayne Metals Research Products Corporation and used without modification. The stent retriever was fabricated by using a 3D braiding technique with a customized braiding machine (provided by Beijing HongHai Microtech Co., Ltd, China) on the shape-setting mold. The heat-setting process was performed at temperatures of 450–550°C for 5–10 min, followed by air cooling. Moreover, stainless steel bands and gilded tungsten wires were braided or twisted with the NiTi mesh as radiopaque markers. The resulting configuration of the NiTi braided mesh (Venus-TD) presented a dumbbell-like geometric shape. This device was then mounted on an internally located core wire that was fixed to the distal end of the stent on one side and on the other to the proximal control handle, which enabled controllable expansion of the Venus-TD with different length/diameter ratios.

### Computational modeling and mechanical evaluation

Numerical modeling of the large elastic–plastic deformation analysis of the stents was performed by using ANSYS Workbench software, which was based on the finite element method using an updated Lagrangian formulation. The non-linear problem that originates from material plasticity and contact constraints was considered by using the Newton‒Raphson approach. The stent models were meshed with 10-node tetrahedral elements. The stent NiTi wires were analysed by using the von Mises plasticity model with a Young's modulus of 67 GPa and a Poisson coefficient of 0.3.

Flat plate compression and three-point bending tests were performed on the Venus-TD to evaluate the radial support force and flexibility, respectively. The analysis was conducted by using a tensile test machine (EUT8201; Shenzhen Sansi Testing Technology Co., Ltd). The circumferential radial resistance force and expansion force were also measured by using a uniaxial test machine (TTR2, Blockwise Engineering LLC).

### Animal and clinical study

All experiments were conducted according to the policies and standards established by our institutional animal research ethics board and all animals were used and managed in accordance with the Guide for the Care and Use of Laboratory Animals (SN2021010). Five experimental miniature pigs weighing 40–45 kg and aged 18 months were included in the experiment. According to the structural characteristics and indications of the device, the IJV was selected as the target vessel for thrombectomy.

Under the guidelines from the European Stroke Organization [42] and the Society of Neurointerventional Surgery [43], a single-center retrospective study including 10 patients diagnosed with CVST was conducted with the Venus-TD (from December 2020 to May 2022). This study was approved by the Ethics Committee of Xuanwu Hospital, Capital Medical University (approval number: 2020037) and was conducted in accordance with the principles of the Declaration of Helsinki. Written informed consent was obtained from all the participants or their direct relatives.

## Supplementary Material

nwaf015_Supplemental_Files
