# Supplementary material for: Dumbbell-shaped thrombectomy device for cerebral venous sinus thrombus removal with controllable axial and longitudinal maneuverability
Source: Natl Sci Rev. 2025 Jan 14;12(3):nwaf015. doi: 10.1093/nsr/nwaf015 (PMC11827591; doi:10.1093/nsr/nwaf015)
Supplement: nwaf015_Supplemental_Files [file nwaf015_supplemental_files.zip › Supplementary data.pdf]

Supplementary Materials for

Title

Dumbbell-shaped thrombectomy device for cerebral venous sinus thrombus removal with controllable axial and longitudinal maneuverability

Authors

Ming Li[1,†], Baoying Song[2,†], Yan Wu[1,2,†], Yang Zhang[1,2,3], Xiaofeng Cao[4], Hongkang Zhang[4], Yi Xu[1,2] Chuanjie Wu[1,2], Chuanhui Li[2], Chen Zhou[3], Lu Liu[2], Feng Yan[2], Sijie Li[1], Jian Chen[2], Ran Meng[2], Jiangang Duan[2], Di Wu[1], Lin Zuo[5], Zikai Xu[6]， Zhou Li[7,*], Yufeng Zheng[4,*], Miaowen Jiang[3,*] and Xunming Ji[1,2,3,*]

Affiliations

[1] China-America Institute of Neuroscience and Beijing Institute of Geriatrics, Xuanwu Hospital, Capital Medical University, Beijing 100053, China;

[2] Department of Neurosurgery and Neuroscience, Xuanwu Hospital, Capital Medical University, Beijing 100053, China;

[3] Beijing Institute of Brain Disorders, Capital Medical University, Beijing 100069, China;

[4] School of Materials Science and Engineering, Peking University, Beijing 100871, China;

[5] School of Bioengineering, Beihang University; Beijing 100191, China;

[6] School of Life Science, University of Glasgow, Glasgow G12 8QQ, Scotland;

[7] CAS Center for Excellence in Nanoscience, Beijing Key Laboratory of Micro-Nano Energy and Sensor, Beijing Institute of Nanoenergy and Nanosystems, Chinese Academy of Sciences, Beijing 100083, China

*__Corresponding authors.__ E-mails: zli@binn.cas.cn; yfzheng@pku.edu.cn; jiangmiaowen415@163.com; jixm@ccmu.edu.cn

†Equally contributed to this work.

This PDF file includes:

Materials and Methods
Figs S1 to S13
Tables S1 to S8

Other Supplementary Materials for this manuscript include the following:

Movies S1 to S7

MATERIALS AND METHODS

Stent manufacture

NiTi wires (50.7 at.% Ni) with a diameter of 0.06 mm were purchased from Fort Wayne Metals Research Products Corporation and used without modification. The stent retriever was fabricated using a 3D braiding technique with a customized braiding machine (provided by Beijing HongHai Microtech Co., Ltd, China) on the shape-setting mold. The heat setting process was performed at temperatures of 450~550℃ for 5~10 min, followed by air cooling. Moreover, stainless steel bands and gilded tungsten wires were braided or twisted with the NiTi mesh as radiopaque markers. The resulting configuration of the NiTi braided mesh (Venus-TD) presented a peanut-like geometric shape. Then, this device was mounted on an internally located core wire that was fixed to the distal end of the stent on one side and on the other to the proximal control handle, which enabled controllable expansion of Venus-TD with different length/diameter ratios.

Computational modeling

Numerical modeling of the large elastic-plastic deformation analysis of the stents was performed using ANSYS Workbench software, which was based on the finite element method and using an updated Lagrangian formulation. The nonlinear problem that originates from material plasticity and contact constraints was considered using the Newton–Raphson approach. The stent models were meshed with 10-node tetrahedral elements. The stent NiTi wires were analyzed using the von Mises plasticity model with a Young's modulus of 67 GPa and a Poisson coefficient of 0.3. The maximum and minimum grid sizes were 0.5 and 0.2, respectively. Two methods were employed to modelling filament interaction in braided stents, the join method and the weave method. The former one features in that the related nodes at wire cross-over points remain in contact and welded; and the weave mode includes the over-under geometry of the NiTi filament and allow both rotation and sliding.

The compression, release, stretch and slide behavior of Venus-TD in venous vessels were also simulated in a jugular vein model (elliptical-like structure with a major axis of 8 mm, minor axis of 7 mm, and thickness of 0.33 mm) and a superslope sinus model (isosceles triangle-like structure with a base of 9.9 mm, height of 6.8 mm, and thickness of 0.8 mm) using Abaqus software. Both of the IJV and SSS vessels were meshed with C3D8R elements. A nonlinear elastodynamic system was used to simulate the blood vessel using the hyperelastic Saint-Venant-Kirchhoff (SVK) model, and it has the following strain energy function:

$$\varphi(\mathrm{F}) = \mu E : E + \frac{\lambda}{2}(tr(E))^2,$$

Where $E = \frac{1}{2}(\mathrm{F}^T\mathrm{F} - I)$ is the Green-Lagrange strain tensor; λ and μ are the Lame constants.

In vitro phantom study

The delivery performance of Venus-TD was tested in an *in vitro* phantom study. A 1:1 venous vascular (from the femoral vein up to the superior sagittal sinus) model was prepared using 3D printing injection molding technology (Shanghai Preclinic Medical Technology Co., Ltd.). The model was filled with 37 ±2℃ saline, and the stent retriever was advanced to the target position along with a Neuromax088 guiding catheter and Stryker 0.014" microwire to test the device flexibility.

Mechanical evaluation

Flat plate compression and 3-point bending tests were performed on the Venus-TD to evaluate radial support force and flexibility, respectively. The analysis was conducted using a tensile test machine (EUT8201; Shenzhen Sansi Testing Technology Co., Ltd.). For the compression test, the stent device was placed between two plate transducers and compressed from 0% to 35% of the labeled diameter at a rate of 20 mm/min. The bending test was performed in a horizontal plane, and the stent was vertically supported by a 20.0 mm wide base plate. The bending force was applied to the middle of the stent by a loading pin with 8 mm deflection at a 20 mm/min displacement rate.

The circumferential radial resistance force and expansion force were also measured by a uniaxial test machine (TTR2, Blockwise Engineering LLC). The stent was fully positioned in the crimping machine, and the initial and final compression station diameters were set at 9 mm and 3.5 mm, respectively. Each sample was cycled four times with an opening/closing speed of 0.02 mm/s. The stent ends were fixated during mechanical testing.

Immersion test

The utilized NiTi wires before and after heat treatment were immersed in simulated body fluid (SBF), with the following ion concentrations: Na^+ 142.0, K^+ 5.0, Mg^{2+} 1.5, Ca^{2+} 2.5, HCO_3^- 4.2, Cl^- 147.8, HPO_4^{2-} 1.0, and SO_4^{2-} 0.5 Mm, which closely mimics human blood plasma. The immersion test was conducted in SBF at 37 ±0.5℃ for 30 days at a ratio of 20 mL/cm^2 and the concentration of released Ni ions was determined by inductively coupled plasma atomic emission spectrometry (Leeman, Profile ICP−AES).

Cytocompatibility analysis

The cytotoxicity of Venus-TD NiTi materials to human umbilical vein endothelial cells (HUVECs) was evaluated using an indirect method according to ISO 10993-5:2009. Both pristine and heat-treated NiTi wires were ultraviolet-radiated for 2 h and incubated in DMEM serum-free medium for 72 h. The cell lines were precultured in 96-well plates with a seeding density of 5×10^3 cells/100 μl per well using Dulbecco’s modified Eagle’s medium (DMEM) supplemented with 10% fetal bovine serum, 100 U mL^{-1} penicillin and 100 μg mL^{-1} streptomycin in a humidified atmosphere with 5% CO_2 at 37℃ for one day. The next day, the culture medium in each well was replaced with prepared extracts supplemented with 10% fetal bovine serum. In the control groups, DMEM was used as a negative control, and 10% DMSO DMEM was used as a positive control. After 24, 72 and 120 hrs of incubation, 10 μl of CCK-8 solution was added to each well for 3 h of incubation. The measurement of the solution optical density (OD) was performed by a spectrophotometer (Elx-800, bio-Tek instruments) at 450 nm wavelength, with a reference wavelength of 630 nm. The cell viability was calculated as follows:

$$\text{Cell viability (\%)}=\frac{OD(test)-OD(blank)}{OD(negative\ control)-OD(blank)}\times 100\%$$

Animal study

All experiments were conducted according to the policies and standards established by our institutional animal research ethics board, and all animals were used and managed in accordance with the Guide for the Care and Use of Laboratory Animals. Five experimental miniature pigs weighing 40-45 kg and aged 18 months were included in the experiment.

To establish device delivery access, it was impossible to enter the brain through the tortuous vertebral vein, and the internal jugular vein (IJV) could only be used to retrograde through the narrow sigmoid sinus to reach the intracranial venous sinus. Approximately half of the pigs had sigmoid sinus dysplasia due to narrowing of extracranial to intracranial access vessels, making it difficult to establish device delivery channels by intervention. According to the structural characteristics and indications of the device, the IJV and common jugular vein (4-6 mm in diameter) of swine are similar to the human venous sinus. The main outcome measures were immediate recanalization and procedural complications (venous sinus perforation, vasospasm, pulmonary embolism, etc.). The venous vessels were stained with hematoxylin and eosin (HE).

Clinical study

Enrollment criteria

A single-center retrospective study including 10 patients diagnosed with cerebral venous sinus thrombosis by imaging examination and treated with endovascular mechanical thrombectomy (March 2018-May 2022) was approved by the institutional review board at Xuanwu Hospital, Capital Medical University. All patients met the following enrollment criteria: 1) 18-70 years of age; 2) confirmed diagnosis of CVST by clinical and imaging examination; 3) an indication for EVT, defined as the presence of one or more severe conditions that are predictors of poor clinical outcome (aggravated conditions despite anticoagulation treatment; severe disturbance of consciousness or altered mental status, such as coma; involvement of multiple venous sinuses leading to severe intracranial hypertension and rapid visual loss; large mass effect lesions, including venous infarction or intracranial hemorrhage; deep venous system and straight sinus thrombosis); and 4) an expected survival time exceeding one year. A standard clinical research form (CRF) was used to record baseline characteristics, including age, sex, clinical and radiological characteristics, NIHSS upon admission, GCS upon admission, symptom onset to operation time, locations of thrombi, procedure time and clinical outcomes.

Outcome measures

The primary clinical outcomes included immediate recanalization grade (complete recanalization: uninterrupted blood flow in the venous system and a small amount of residual thrombus in the vessel wall; partial recanalization: quantities of thrombus residue detected, intermittent venous blood flow interruption; no recanalization: interruption of blood flow in the venous system). Patients recovered without a disability was defined as a modified Rankin scale score (mRS) of 0-1 at 90 days. Other treatment-related parameters included procedure time (from device implantation to the last thrombectomy operation and then withdrawal of the device), intracranial circulation time (from the first appearance of the contrast agent in the arterial system to the complete clearing from the venous system), procedural complications and device-related adverse events.

Thrombectomy device and technique

Under the guidance of the route map, one side of the IJV (mostly the lesion side) was punctured, and an 8F sheath was inserted. The guide catheter was retrogradely advanced into the venous sinus, and the Venus-TD was delivered to the distal end of the venous sinus thrombus along the micro-guidewire. Then, the thrombectomy device was released and adjusted according to the diameter of the lumen. Through radial diameter change and axial movement of the thrombectomy net, the thrombus was mechanically fragmented

and removed from vessels, during which negative pressure was used to suction the thrombus fragments by Autotransfusion System (Autolog, Medtronic, Inc, Minneapolis, Minnesota; as shown in Fig.S7). The retrieved thrombus was stained with hematoxylin and eosin (HE).

MRBTI imaging protocol and evaluation

All MR studies were conducted on a 3.0T system (MAGNETOM Verio, Siemens Healthcare, Erlangen, Germany) using a 32 - channel head coil for signal reception. Typical imaging parameters for MRBTI included: repetition time (TR) = 600 msec, echo time (TE) = 14 msec, spatial resolution = 0.8 × 0.8 × 0.8 mm3, scan time = 5 min. All MRBTI images were blindly reviewed by two experienced neuroradiologists. The ratio of the signal intensity within each venous sinus to the standard deviation of the background noise was used to identify the thrombus. The volume of the thrombus was quantitatively measured on MRBTI images, both before and after endovascular intervention, utilizing commercial software (Vessel Analysis, China).

Statistical analysis

Statistical analysis was conducted using a one-way analysis of variance (ANOVA), followed by Tukey's post hoc tests within SPSS version 19.0, where p-values below 0.05 were deemed statistically significant. All continuous variables are presented as the means ± SDs and median.

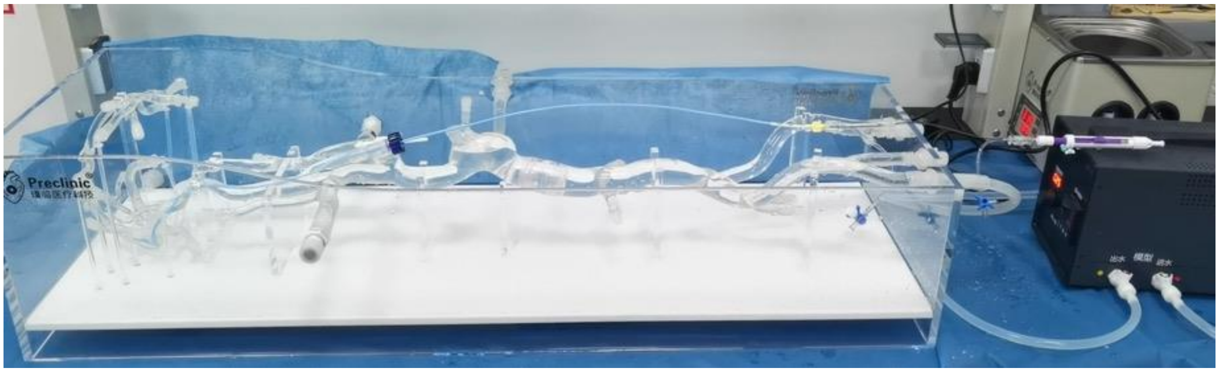

Figure S1.

Photos of the 1:1 venous vascular 3D model for evaluating the delivery performance of the Venus-TD

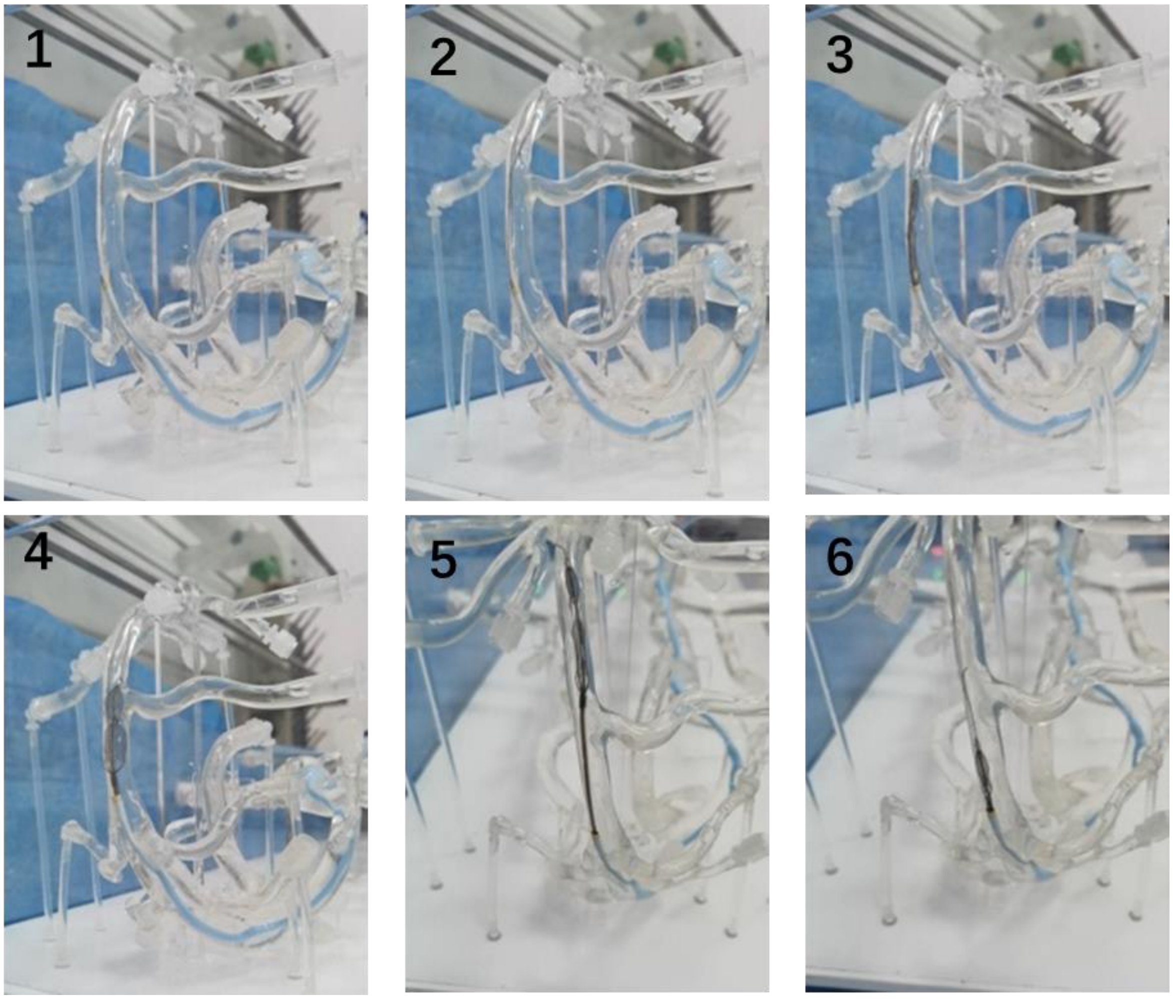

Figure S2.

Delivery/retrieval of Venus-TD into/from the SSS models

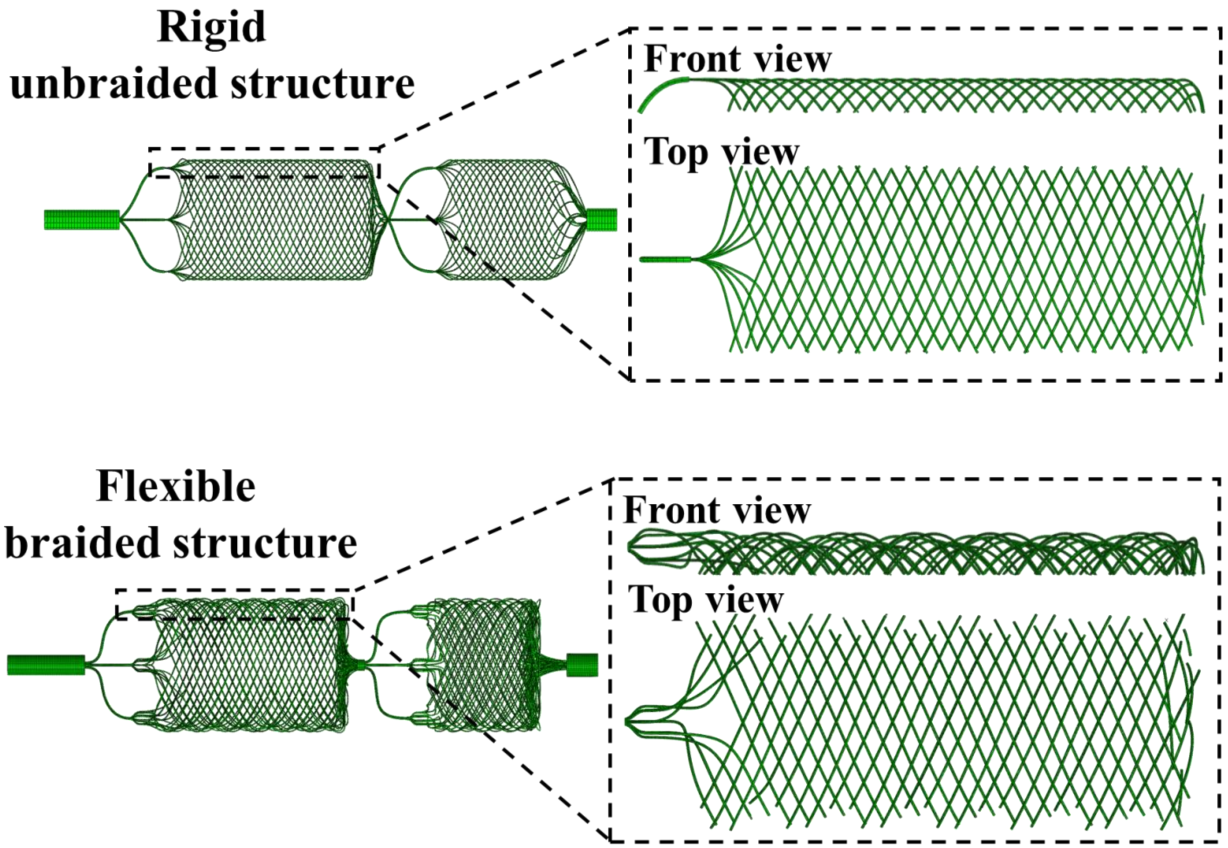

Figure S3. Stent models with join-configurational unbraided and weave-configurational braided structures

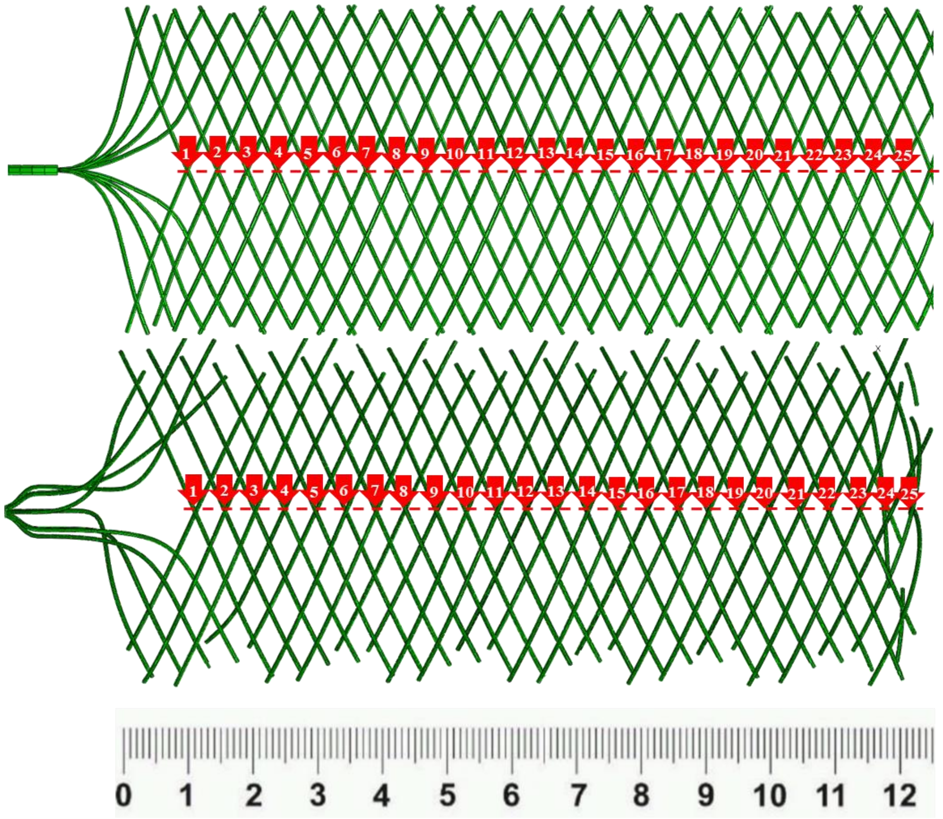

Figure S4.

The point position that being quantitively analyzed by computational modelling

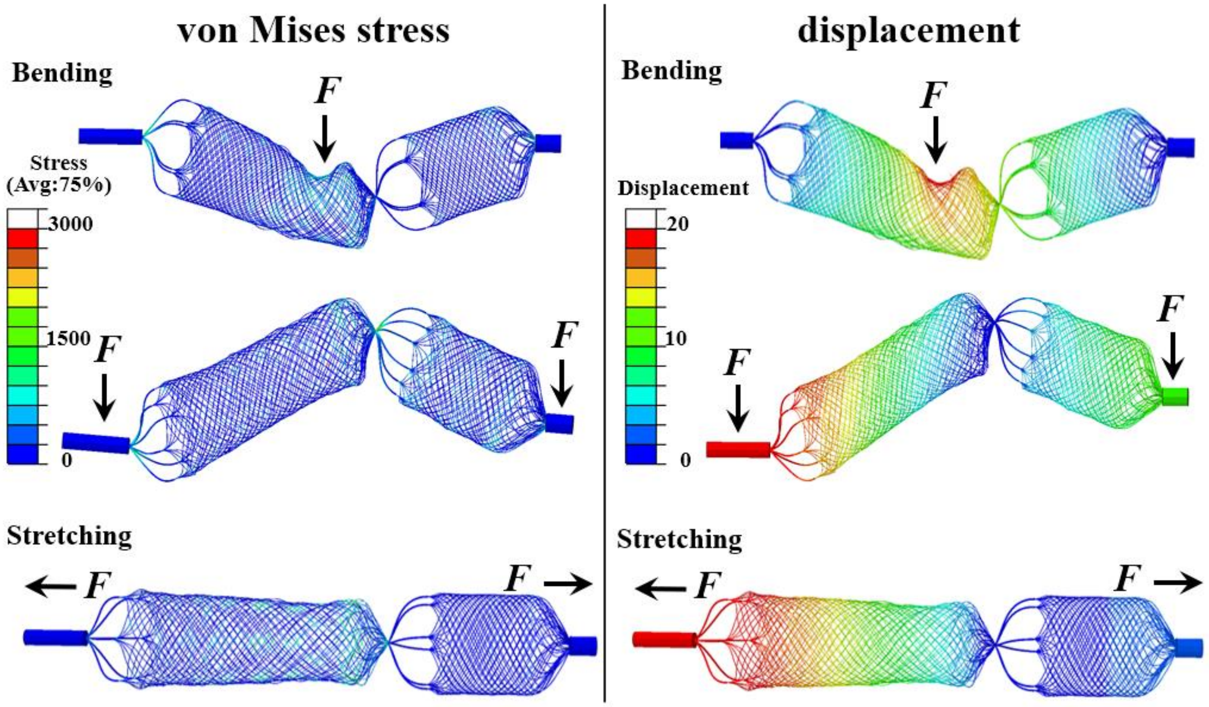

Figure S5.

The von Mises stress and displacement contour plots of the join configuration devices under bending and stretching

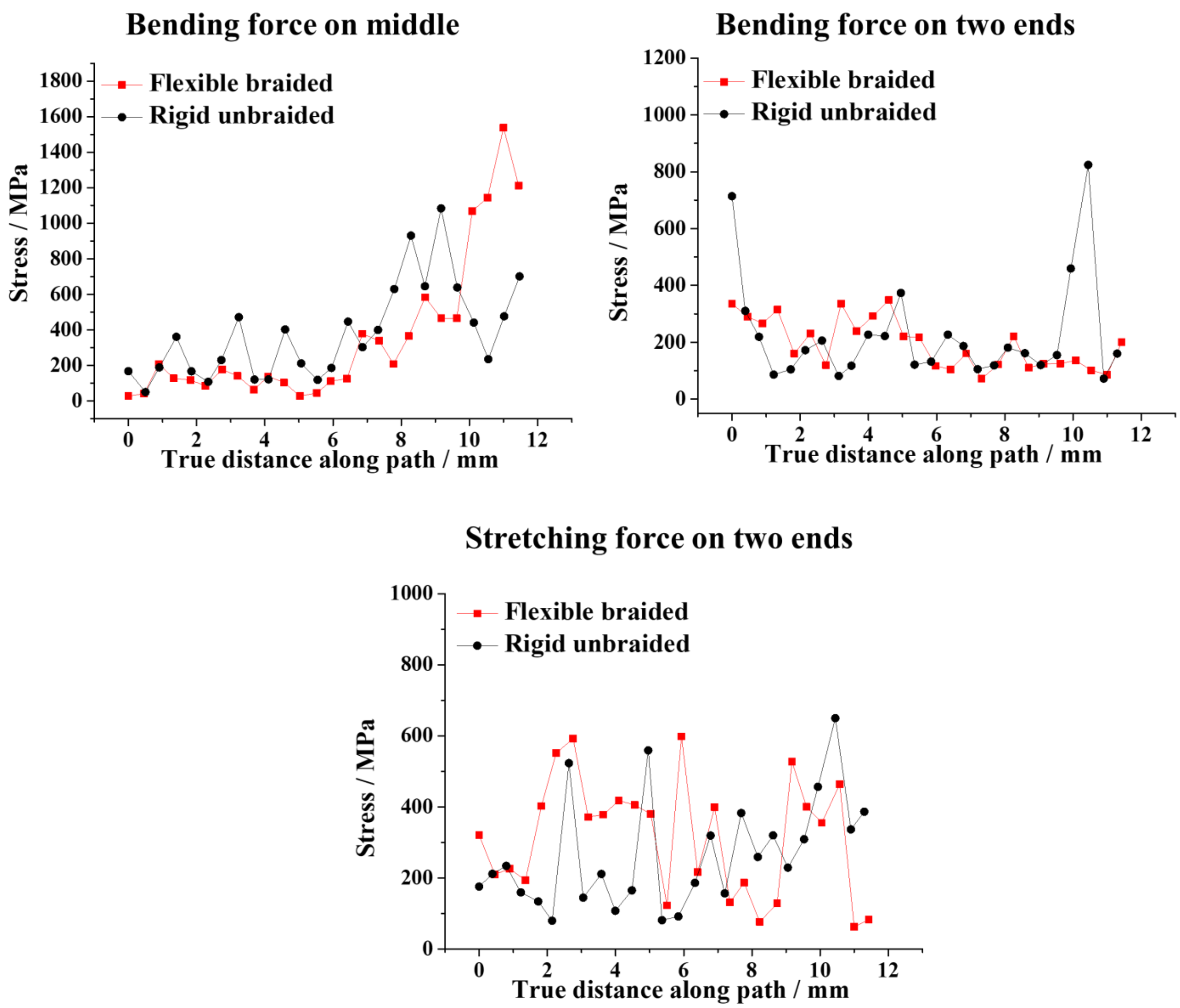

Figure S6.

The von Mises stress of the stents under bending and stretching

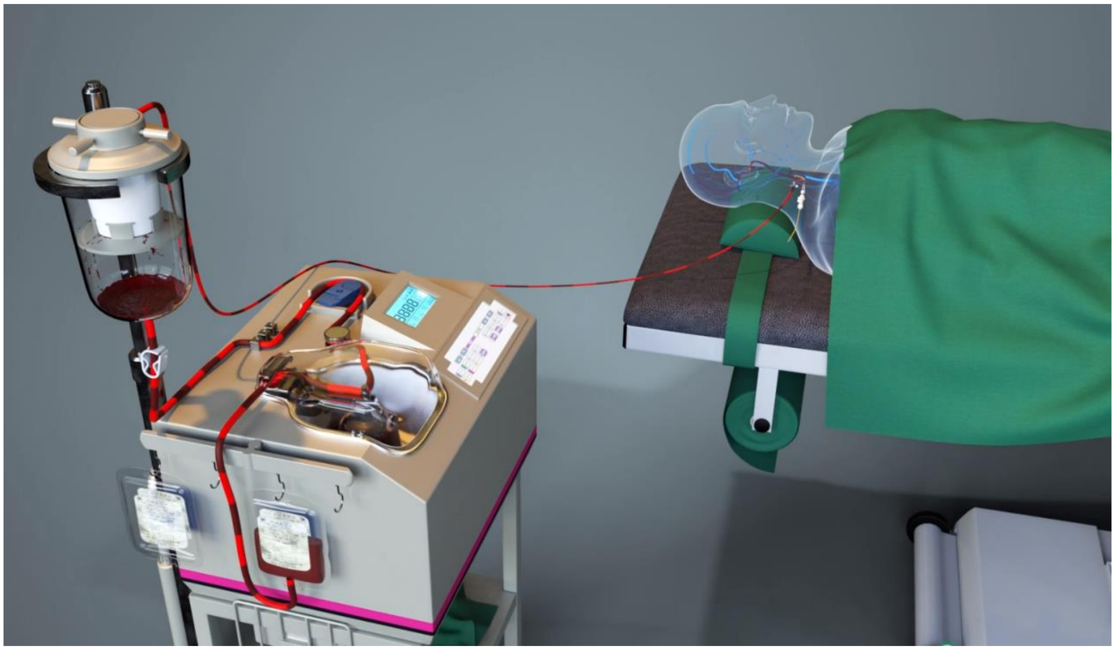

Figure S7.
Illustration of thrombectomy together with aspiration process by using an autotransfusion system

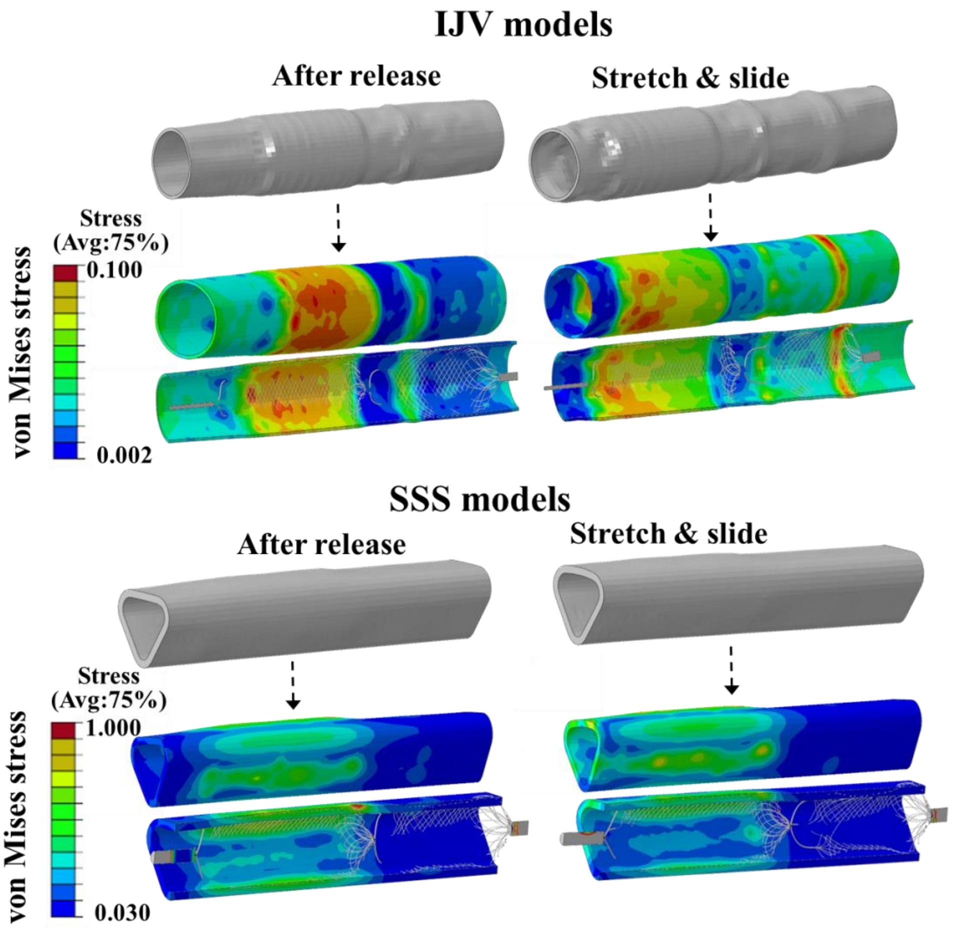

Figure S8.

The von Mises stress distribution on IJV and SSS vessels walls induced by the expand and stretch/slide of the join configuration stent retriever.

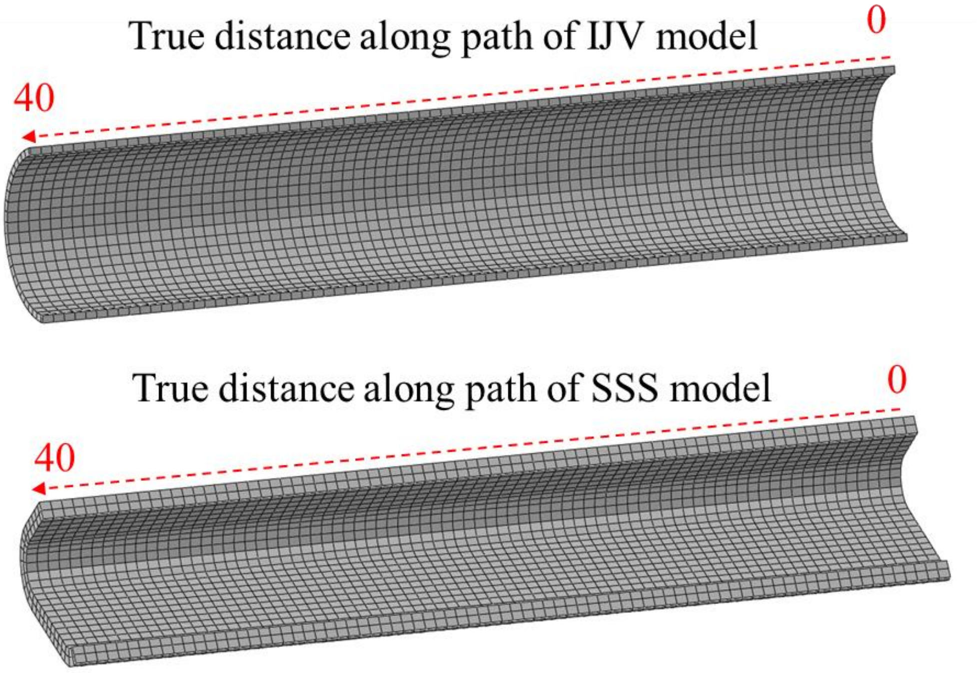

Figure S9.
The point position that being quantitively analyzed by computational modelling

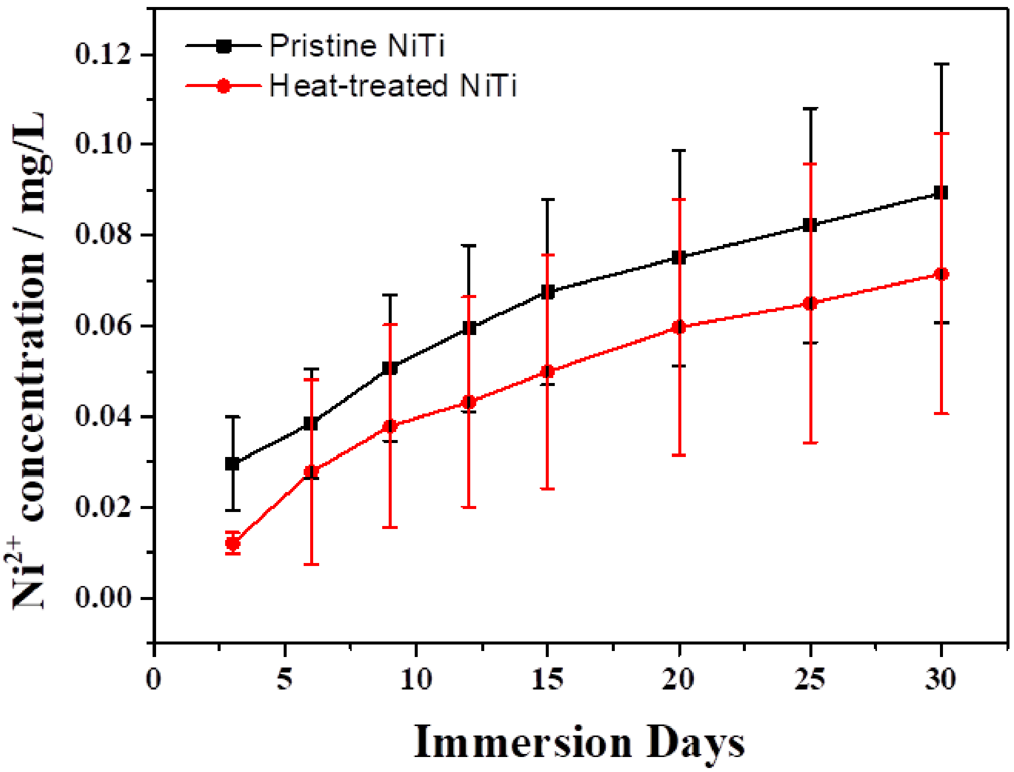

Figure S10.
The release profile of Ni^{2+} in SBF

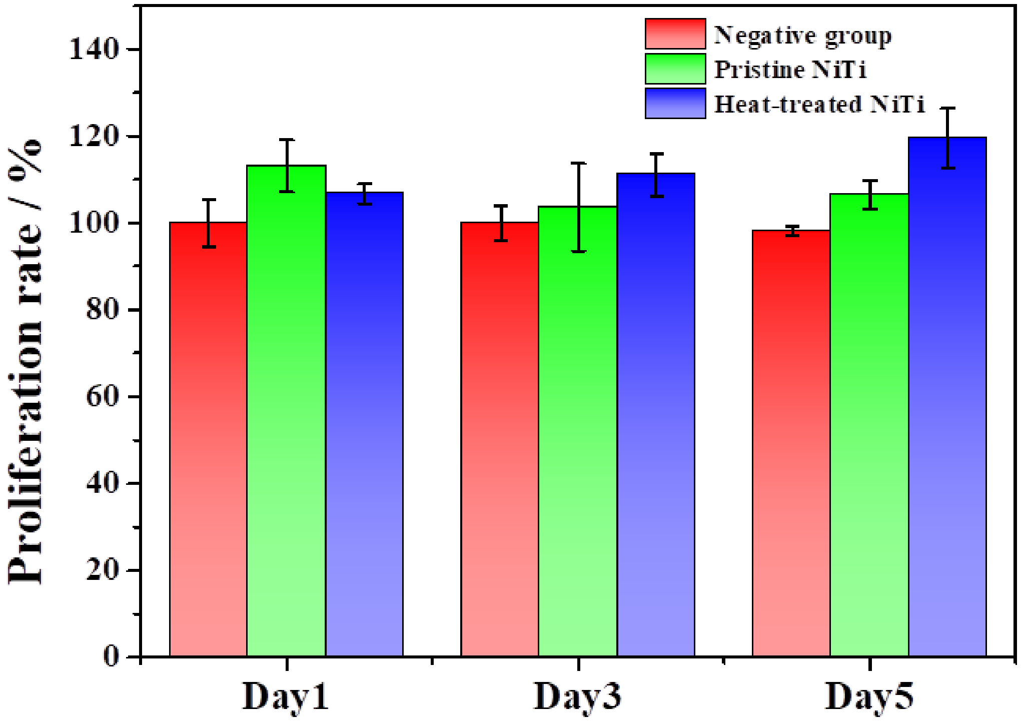

Figure S11.

The proliferation rate of HUVECs in NiTi extract after being incubated for 1, 3 and 5 days

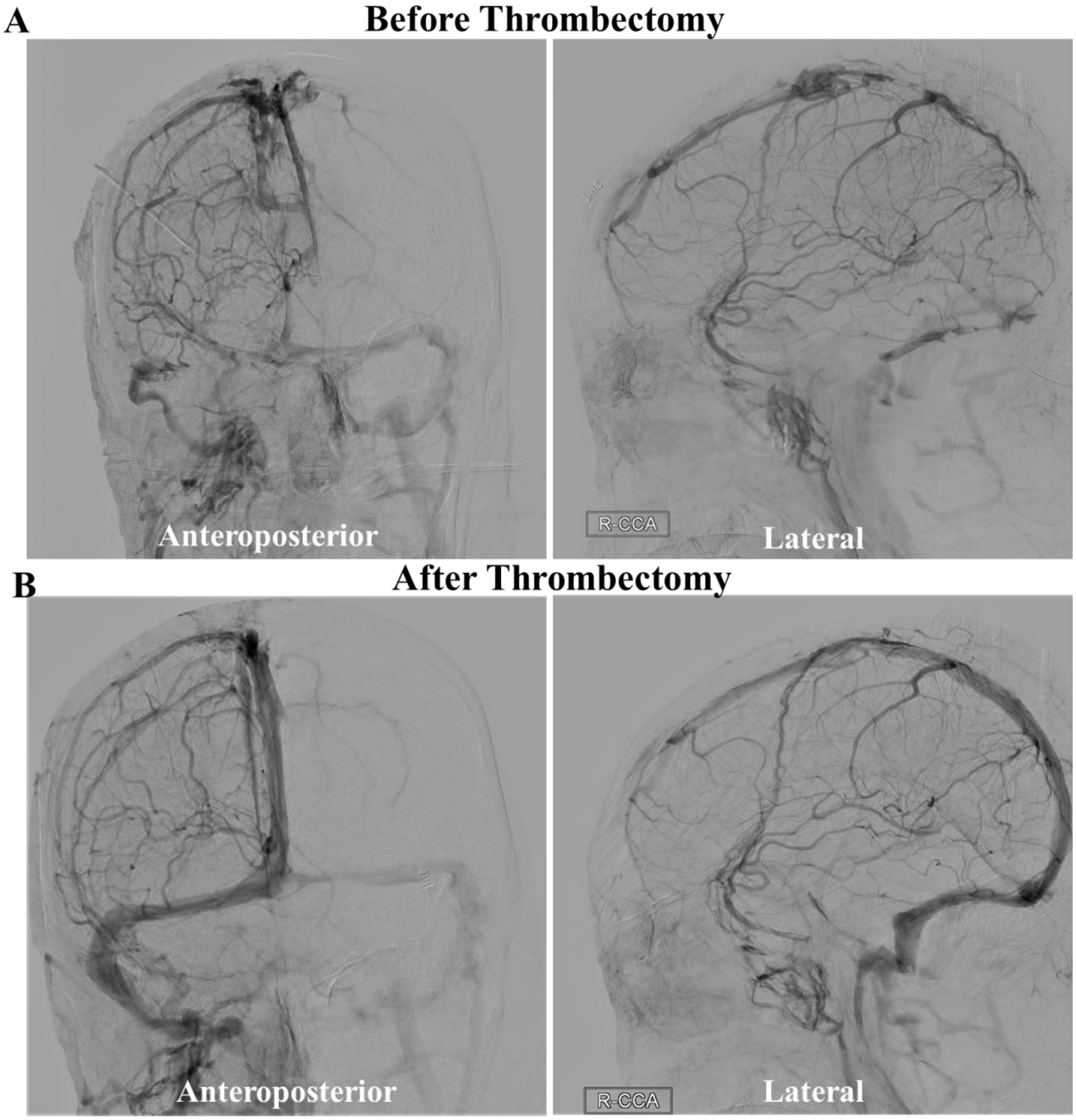

Figure S12.
Venous phase of cerebral DSA before and after the thrombectomy.

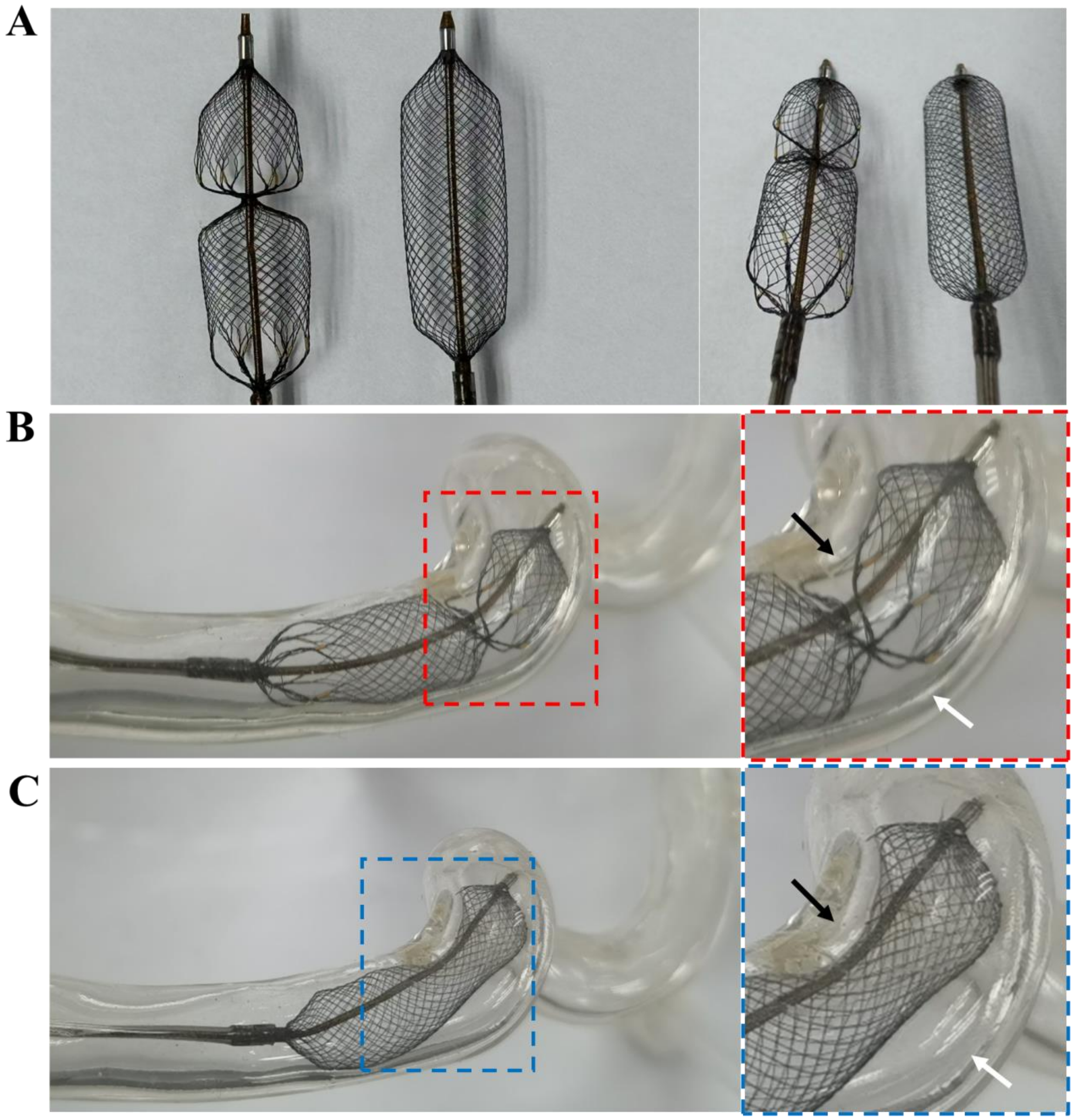

Figure S13.

The comparison of Venus-TD and the control stent retriever with the same length and diameter but without dumbbell structure (A). The demonstration of Venus-TD (B) and the control stent (C) that placed in the sigmoid sinus model. The black and white arrows indicated the interaction of the stent with the vessel walls, suggesting that the device with dumbbell configuration was more mechanically flexible in passing through the vessels with acute anatomic curvature.

Table S1.

Anatomical differences exist between cerebral artery and venous

	Cerebral artery	Cerebral venous
Lumen diameter	Small (2 to 3 mm proximally)	Large (average diameter of SSS is 8mm)
Vessel structure	Have no external elastic lamina, with well-developed internal elastic lamina	Thin smooth muscle layer
Inner vascular surface	——	Trabecular, septal and arachnoid granule structures
Blood pressure	High	Low
Blood flow rate	Fast	Slow
Thrombus composition	Mainly composed of platelets and a small amount of fibrin	Consist of fibrin and red blood cell and a small amount of platelets

Table S2.

The diameters of selected cerebral venous and artery vessels

	Vessels	Diameter Location	Value /mm	Ref.
Cerebral venous	Internal jugular vein	Right	9.1	[44]
		Left	7.4	
	Sigmoid sinus	Right	6.5	
		Left	5.3	
	Transverse sinus	Right	6.4	
		Left	5.4	
	Superior sagittal sinus	Bregma	6.2	[37]
		Midpoint	9.1	
		Lambda	9.4	
Cerebral artery	Internal carotid artery	-	4.66	[45]
	Middle cerebral artery	pM1 Right	2.4	[46]
		pM1 Left	2.3	
		dM1 Right	2.3	
		dM1 Left	2.2	
		M2 Right	1.6	
		M2 Left	1.6	
	Anterior cerebral artery	pA1 Right	1.9	
		pA1 Left	2.0	
		dA1 Right	1.7	
		dA1 Left	1.8	
		A2 Right	1.6	
		A2 Left	1.7	

Table S3.

Parameters of available stent retrievers [47]

No.	Stent	Length	Diameter
1	Solitaire	20	4
2		40	4
3		20	6
4		24	6
5		40	6
6	Trevo XP	20	3
7		20	4
8		30	4
9		25	6
10	ERIC	15	3
11		20	3
12		24	4
13		30	4
14		44	6
15	EMBOTRAP II	21	5
16		33	5
17	CATCH+	15	3
18		20	4
19		30	6
20	APERIO	28	3.5
21		30	4.5
22		40	4.5
23		40	6
24	pREST	20	3
25		20	4
26		20	4
27		30	6
28	TIGERTRIEVER	20.5	2.5
29		23	3
30		32	6

Table S4.

National Institute of Health Stroke Score (NIHSS)

Score	Stroke Severity
0	No stroke symptoms
1-4	Minor stroke
5-15	Moderate stroke
16-20	Moderate to sever stroke
21-42	Sever stroke

Table S5.

Glasgow coma score (GCS)

Behavior	Response	Score
Eye opening response	Spontaneously	4
	To speech	3
	To pain	2
	No response	1
Best verbal response	Oriented to time, place, and person	5
	Confused	4
	Inappropriate words	3
	Incomprehensible sounds	2
	No response	1
Best motor response	Obeys commands	6
	Moves to localized pain	5
	Flexion withdrawal from pain	4
	Abnormal flexion (decorticate)	3
	Abnormal extension (decerebrate)	2
	No response	1
Total score	Best response	15
	Comatose client	8 or less
	Totally unresponsive	3

Table S6.

The recanalization rate of occluded CVST and operation time for the thrombectomy procedure by using Venus-TD and other devices

No.	Author	Time/ min	Treatment	Device	Ref.
1	Our study (Venus-TD)	33.2 ±10.6	Mechanical treatment	Venus-TD	-
2	Peng et al.2021	260	Mechanical treatment	Solitaire FR; Intermediate catheter aspiration	[27]
3	Medhi et al.2020	111	Mechanical treatment plus local thrombolysis or not	Large-bore aspiration catheters	[28]
4	Mera et al.2022	89	Mechanical treatment	Vacuum aspiration thrombectomy	[29]
5	Jankowitz et al. 2013	97 ±17	Mechanical treatment plus local thrombolysis or not	Penumbra aspiration	[30]
6	Lee et al.2016	73.5 ±24.7	Mechanical treatment	Penumbra aspiration; Sterling or Ultrasoft SV	[31]
7	Mortimer et al.2013	210	Mechanical treatment plus local thrombolysis or not	Maceration of thrombus with Guidewire; Penumbra aspiration; Angioplasty; Balloon catheter	[32]
8	Zhang et al.2008	123 ±52	Mechanical treatment plus local thrombolysis	AngioJet; Balloon catheter	[33]
9	Choulakian et al.2010	121	Mechanical treatment	Penumbra aspiration with or without Balloon catheter	[34]
10	Coutinho et al.2020	117	Mechanical treatment	Angiojet; Stent retriever; Balloon catheter; Penumbra aspiration; Fogarty catheter	[5]

Table S7.

Baseline characteristic. NISHSS-national institute of health stroke score, GCS- Glasgow coma score

Number of patients	10
Age year	
Mean ± SD	39.1 ±14.6
Median(range)	38.5(19-67)
Male sex, %(n/N)	60.0(6/10)
Clinical and radiological characteristics	
Headache, %(n/N)	100(10/10)
Nausea and vomiting, %(n/N)	90.0(9/10)
Epileptic seizure, %(n/N)	40.0(4/10)
Blurred vision, %(n/N)	40.0(4/10)
Coma, %(n/N)	40.0(4/10)
Mental status disorder, %(n/N)	10.0(1/10)
Focal neurological dysfunction, %(n/N)	10.0(1/10)
Cerebral venous infarction, %(n/N)	60.0(6/10)
Intracranial hemorrhage, %(n/N)	20.0(2/10)
Subarachnoid hemorrhage, %(n/N)	10.0(1/10)
Admission NIHSS, median(range)	0 (0-21)
Admission GCS, median(range)	15(11-15)
Symptom onset to operation time(day), mean ±SD	21.8 ±12.9
Preprocedure anticoagulation, %(n/N)	70.0(7/10)
Thrombosed sinuses	57
Superior sagittal sinus thrombosis	10(57)
Straight sinus	3(57)
Torcular herophili	7(57)
Transvers sinus	17(57)
Sigmoid sinus	17(57)
Jugular vein	3(57)

Table S8.
The baseline and residual thrombus volume of each segment of 10 cases quantitatively measured on MRBTI images.

Cases	Before thrombectomy	After thrombectomy	Thrombus volume (mm^3)							
			Superior sagittal sinus		Transverse sinus		Sigmoid sinus		Internal jugular vein	
			Baseline	Residual	Baseline	Residual	Baseline	Residual	Baseline	Residual
Case 1	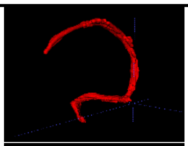	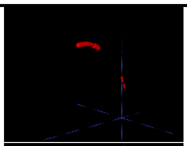	10240	506	3700	41	1940	0	1960	0
Case 2	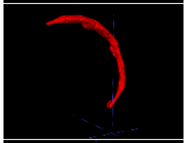	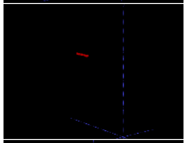	7706	225	NA	NA	NA	NA	NA	NA
Case 3	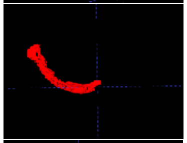	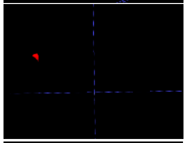	NA	NA	15360	383	NA	NA	NA	NA
Case 4	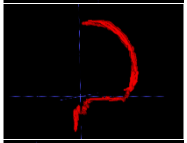	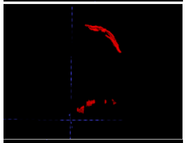	9546	799	2164	292	1740	303	1840	57
Case 5	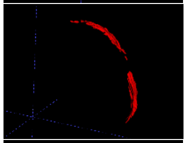	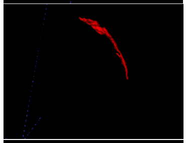	1951	815	NA	NA	NA	NA	NA	NA
Case 6	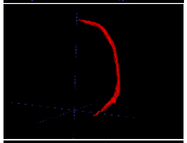	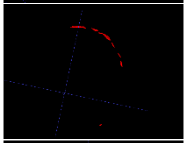	4144	299	222	0	NA	NA	NA	NA
Case 7	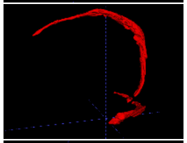	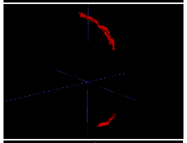	7523	1166	2477	462	1260	141	1550	0
Case 8	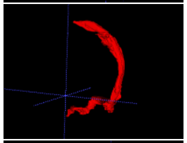	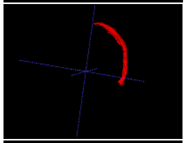	10220	4602	5720	2215	3980	697	2290	787
Case 9	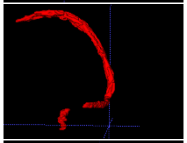	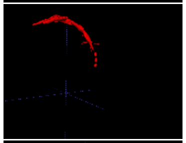	9536	3615	1354	923	980	186	950	626
Case 10	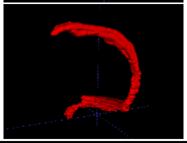	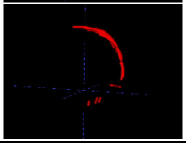	10670	3820	4950	563	1370	101	1210	76

Movie S1.

Animation of the thrombectomy process in CVST

Movie S2.

Video of thrombectomy process in IJV produced by CFD

Movie S3.

Video of thrombectomy process in SSS produced by CFD

Movie S4.

DSA video of thrombectomy process in swine model

Movie S5.

Video of the venous phase of cerebral angiography before thrombectomy

Movie S6.

Video of the venous phase of cerebral angiography after thrombectomy

Movie S7.

DSA video of thrombectomy process in SSS of CVST patient
